# Male Genital Mutilation in the Name of Ritual Circumcision: A Case Report and Literature Review

**DOI:** 10.1155/2023/9935247

**Published:** 2023-10-07

**Authors:** Latif Dar, Alhareth Baarimah, Saeed Alshehrani, Alhassan Alasiri, Mohammad Alassiri, Saleh Al-Ghamdi

**Affiliations:** ^1^Department of Pediatric Urology, Abha Maternity and Child hospital (AMCH), Abha, Saudi Arabia; ^2^Department of Pediatric Urology, Khamis Mushayt Maternity and Children Hospital (KMMCH), Khamis Mushayt, Saudi Arabia

## Abstract

Unlike female genital mutilation, the alteration of male genitals has not received much attention. Circumcision is the most common and oldest surgical procedure being performed. When performed by surgeons or well-trained personal the procedure is safe, but most of the times it is being performed by untrained people with no or little medical background. This has led to many complications. Total skin loss is an uncommon but serious complication. There is an ongoing debate regarding the management of this complication. Here, we present a case of total penile skin loss which had resulted from penile mutilation in the name of ritual circumcision.

## 1. Introduction

Male genital mutilation (MGM) has not received the attention it deserved. Circumcision is unarguably the most common and the oldest surgical procedure being performed [1, 2]. There is an ongoing debate regarding the merits and demerits of the procedure [3–5]. Commonly, the procedure is performed on religious and cultural grounds. If performed by pediatric urologists, surgeons, or well-trained, qualified personnel, the procedure is quite safe, but most of the time it is being performed by untrained people with no or little medical background. This has led to serious complications and even death. Total skin loss is an uncommon but a serious complication [6–8]. There is also a debate regarding the way this complication is being repaired [9, 10]. Here, we present a case with total penile skin loss resulting from penile mutilation in the name of ritual circumcision.

## 2. Case Presentation

A six-year-old boy had undergone penile mutilation in the name of ritual circumcision at the hands of his grandfather. Using his traditional knife, the grandfather degloved his penis by the guillotine method. No local anesthetic was used. This was followed by the application of some locally available herbs thought to control the bleeding, but this did not stop the bleeding. The poor boy continued to bleed from his degloved penis. In the evening, he was taken to a hospital where the doctors applied a dressing to control the bleeding. Later in the day, the patient was referred to our hospital.

On arrival at the ER, the patient was in a state of shock. He was in pain, ill-looking, pale, and tachypneic. His blood pressure was 80/40, his heart rate was 140/min, and his respiratory rate was 24/min. He had not passed urine since the event.

**Figure 1:**
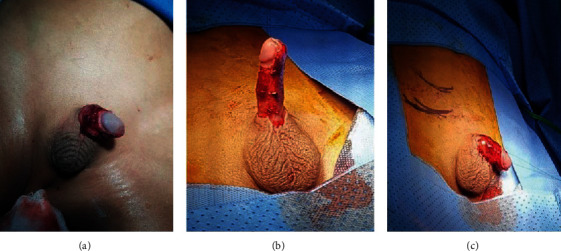
Preoperative assessment of the damage and skin loss.

A local examination revealed blood-soaked gauze pads which were applied over his perineum; the underlying dressing was completely soaked in blood. The dressing was removed, and the wound was cleaned with saline. There was total loss of penile skin with multiple bleeding spots over the shaft. Glans was intact, and both testes were in place. The bleeding was controlled using pressure dressing. The patient received fluid boluses. A broad-spectrum antibiotic (ceftriaxone 75 mg/kg I.V.) and analgesia (paracetamol 10 mg/Kg I.V) were given. Labs were drawn. His hemoglobin was 10 g/dl and his hematocrit was 30%. The rest of the labs was within normal limits (Figure 1).

**Figure 2:**
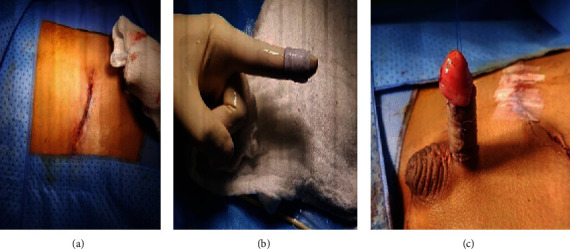
Full-thickness skin grafts (FTSG) from both the groins being harvested and defect repaired.

After resuscitation, the patient was shifted to the operating room. Under general anesthesia, the patient was reassessed. Foley's catheter was put in. The skin loss was reassessed and found to be too large to be covered by the scrotal skin mobilization. Since the scrotum at this age is small, it was not advisable to use a scrotal skin flap. It was decided to take full-thickness skin grafts from both the groins. The grafts were harvested. The graft bed over the shaft of the penis was prepared; it was looking well perfused. One of the grafts was applied over the dorsum of the penis using 6-0 PDS anchoring sutures. It covered almost the two-thirds of the shaft. A second graft was used to cover the remaining defect using the same sutures. The graft was meshed, and a good circumferential dressing was applied. Graft sites were primarily closed (Figure 2).

Post operatively, the patient behaved well. The dressing was cautiously removed on the 6^th^ post op day. Both the grafts were well taken and were looking healthy. The next day, the catheter was removed, and the patient was discharged home.

**Figure 3:**
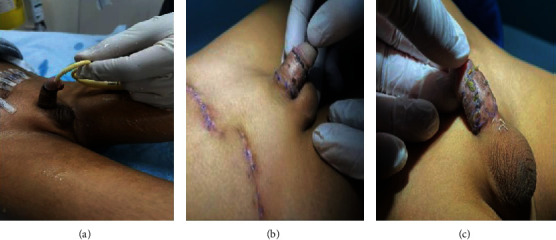
Post operative appearance. (a) At 10^th^ post op day. (b, c) Six weeks of operation.

At the one-month follow-up, the patient was doing well, and the family was happy with the final results (Figure 3).

## 3. Discussion

Circumcision is unarguably the most common and oldest surgical procedure being performed [1]. There is an ongoing debate regarding male circumcision. Arguments have been put forth both for and against the procedure. It has some proven advantages like decrease in incidence of sexually transmitted disease and HIV in circumcised men. More than 40 observational studies have reported a protective effect of 40% to 88% against HIV acquisition in circumcised sexually active men [2–4]. In their meta-analysis of fifteen studies, Lei et al. found a 70% protective effect of circumcision against HIV acquisition [4]. Studies have also shown a decrease in incidence of invasive penile carcinoma in circumcised men; however, the data is not conclusive to recommend male circumcision as a preventive strategy in this regard [5]. It has also an advantage of good hygiene and decreased incidence of balanoposthitis. There is also exists a sizable opinion against the procedure. Some consider it a male genital mutilation (MGM), which by definition is a temporary or permanent modification of external genitalia that involves partial or total ablation of genital tissues or other injuries to the male genital organs [6].

When performed by pediatric urologists, surgeons, and properly trained professionals, circumcision is a safe procedure with a complication rate of 0.2 to 5%. Most of these complications are minor such as bleeding, infection, swelling, and meatal stenosis [2, 3]. Most of the circumcisions are performed for religious and cultural reasons and are performed by people with little or no medical background. It is under these circumstances that it becomes MGM in the true sense of the term [3, 7]. Under these settings, there is not only an increase in the rate of complications but also the complications become more serious and dreadful. Ritual circumcision has become synonymous with complications in rural and tribal areas of Africa and other developing countries. Serious complications like amputation of the penis, glans amputation, urethro-cutaneous fistula, partial or total loss of penile skin, and severe hemorrhage resulting in shock and death of the victims have been reported with traditional and ritual circumcision [1, 3, 8].

These complications are avoidable or at least can be brought to the minimal possible level by providing well-trained professionals at the facilities offering circumcision, especially in rural areas. No country in the world has banned circumcision, but many countries have legislations which regulate the practice of circumcision. These laws require circumcision to be performed by well-qualified, trained professionals in the facilities with proper sterilization, wound care, and anesthesia. Under these conditions, complications are rare [10].

Our patient was literally butchered by his grandfather in the name of ritual circumcision. He had lost all his penile skin. Even after this horrific incident, the family had not sought medical help, and he continued to bleed. It was only when his condition became very serious that he was taken to a hospital. Despite having state-of-the-art medical facilities all around the Kingdom of Saudi Arabia where neonatal circumcision is being performed by doctors, these tribal people continue their traditional practice of ritual circumcision.

Total loss of penile skin is rarely seen in medical practice. If not attended in time and managed properly, it may lead to permanent genital deformity. It may also result in severe hemorrhage and death of the patient [2, 3].

Repair of total penile skin loss poses a challenge to the attending surgeon. Mobilizing the scrotal skin and repairing the defect frequently leads to buried and trapped penis [6]. The scrotal flap has good cosmetic and functional results. It needs sufficient scrotal skin which is available only in case of adult and adolescent patients. In infants and children, the scrotum is not well developed to allow sufficient skin to be used as a flap. Facio-cutaneous advancement flaps from the inguinal and femoral regions have also been used, the disadvantage being the bulky appearance of the penis. A split skin graft is vulnerable to secondary contraction. It allows a thin covering which is susceptible to trauma and ulcerations, especially during sexual intercourse [9].

Full-thickness skin graft (FTSG) goes through primary contraction, but long-term cosmetic and functional results are good. FTSG from the groin has the advantage of being nonhirsute, allowing good cosmetic appearance, and having enough elasticity to allow normal erectile function and sexual activity [9].

We used bilateral groin flaps as the defect was large and a single graft would have created a large graft site defect not amenable to primary closure. The graft sites were primarily closed, and the healing was good with an acceptable cosmetic appearance.

## 4. Conclusion

Ritual circumcision when performed by untrained people with little or no medical background results in serious complications. Our case report emphasizes the need to legally regulate the practice of circumcision and provide for training of rural practitioners to reduce the incidence of complications. Repairing total skin loss is a challenge for surgeons, FTSG is a good alternative for the repair of complete penile skin loss. It has the advantage of being hairless, has good elasticity, and has a good cosmetic and functional outcome.

## Data Availability

Data is available.
